# The Sensory Phenomena of Tabes Dorsalis

**Published:** 1908-10-17

**Authors:** 


### Neurology.

### THE SENSORY PHENOMENA OF TABES DORSALIS.

The sensory phenomena to be noted in cases of locomotor ataxia are of three separate kinds, namely:—

1. Subjective pains, such as lightning pains in the legs and gastric and other crises.

2. Subjective derangements of sensation of which no objective proof can be obtained, such as the cotton-wool feeling in the soles of the feet.

3. Derangements of sensation, sometimes not complained of spontaneously by the patient, but detectable objectively in the form of areas of impaired cutaneous sensibility.

1. *Lightning and Other Pains.*—These are sharp, shooting, or stabbing pains which come on in paroxysms during which each individual pain is of momentary duration. The paroxysms may last for minutes only, or for several consecutive hours, after which there may be a considerable interval before the next paroxysm. These lightning-like neuralgic pains are eminently characteristic of locomotor ataxy, being by far the most frequent of all the manifestations of the disease, and in many instances a comparatively early sign.

The pains are practically always felt in the legs, whether they occur in other parts as well or not. They are next most frequent in the trunk, either with or without visceral symptoms, such as vomiting or tenesmus. The so-called "crises," such as a gastric crisis, are precisely analogous to lightning pains in the legs. In each case the underlying pathology is the subacute inflammation and degeneration of the posterior nerve roots and their ganglia, the lesion in which causes pain just as it does in herpes zoster. Irritation of the substance of the spinal cord itself causes no pain, but similar irritation of the posterior nerve roots leads to intense pain. In locomotor ataxy these roots are affected earlier and to a much greater extent in the lumbar region of the cord than they are higher up—a fact which explains why lightning pains in the legs are so much commoner than are gastric or other visceral crises.

A not very uncommon error of diagnosis is to mistake the lightning pains of tabes for those of chronic rheumatism or perhaps lumbago or sciatica. One reason for this may perhaps be that they are apt to be influenced by weather a good deal, being worse during wet, damp, or thundery weather, and particularly at night. Another condition for which they may be mistaken is that of alcoholic peripheral neuritis, the absence of knee-jerks being common to both diseases. In locomotor ataxy, however, there is no actual loss of strength in the legs, although, from inco-ordination, the patient may say he is too weak to walk. Even a bedridden tabes patient still has good muscle power in the legs, whereas peripheral neuritis causes absolute weakness. This means of distinction is a very important one, because the absence of knee-jerk is common to both diseases, and in alcoholic subjects the pupil reactions are often so abnormal that it may be difficult to decide whether they are of the Argyll-Robertson type or not; in certain cases the differential diagnosis between locomotor ataxy and peripheral neuritis is decided entirely by testing the degree of muscle power the patient still retains. The importance of gastric crises is that they may be mistaken for perforated gastric ulcer, a laparotomy having more than once been performed unnecessarily owing to this mistake. In regard to treatment, drugs of the antipyrine group give the greatest relief. Aluminium chloride in two-grain doses in pill form has been accredited with giving good results sometimes. The one thing to avoid is morphia; the very fact that it relieves the pains induces repetition of the injection, and hence the morphia habit. Fortunately, although lightning pains are an early sign, they are also one which very often disappears spontaneously as the ataxy becomes more marked.

2. *The entirely subjective derangements of sensation* of which tabetic patients complain are well enough known. The most frequent is a feeling in the soles of the feet as if the patient were walking upon a cushion or upon soft feathers or cotton-wool. Other forms of the symptom are numbness, formication, tingling, a feeling of coldness, or hyperæsthesia or increased sensibility of the skin. None of these are very troublesome as a rule, or at any rate they can be supported by the patient without any particular treatment.

3. *The objective derangements of sensation* are perhaps not sufficiently insisted upon as a rule; they may be of considerable diagnostic significance, especially in the earlier stages of tabes dorsalis, helping to distinguish it from other forms of ataxia in which anæsthesia does not occur. In nearly every case of locomotor ataxy it is possible to demonstrate some impairment or loss of sensibility to touch, or of the sensibility to pain, or of the sensibility to heat and cold in some part or other of the body. The distribution of the paræsthesia or of analgesia is usually quite irregular. Analgesia develops earliest as a rule in these cases, and its most common sites are the front of the thorax, the abdomen, the inner sides of the upper arms, the outer sides of the legs, the backs of the calves, and the soles of the feet. There is no place that may not exhibit the impaired sensibility, however, and one can only say that the head, face, and neck are the parts least often affected by it. In many cases of locomotor ataxy one finds a band of analgesia, and sometimes also of impaired cutaneous sensibility or of impaired sensibility to hot and cold stimuli in what may be termed the thoracic, the abdominal, and the ulnar bands. Dr. Byrom Bramwell points this out, laying stress upon the diagnostic value in the early stages of the disease, of such a band of impaired sensibility around the thorax, usually below the level of the third rib, or around the upper part of the abdomen, or down the ulnar side of upper arm and forearm on one side or upon both. Absence of paræsthesia does not exclude tabes dorsalis, but presence of it often assists in clinching an early diagnosis.

